# Microleakage and penetration capability of various pit and fissure sealants upon different sealant application techniques

**DOI:** 10.4317/jced.60577

**Published:** 2023-10-01

**Authors:** Apa Juntavee, Niwut Juntavee, Achara Chaisuntitrakoon, Phillip L. Millstein, Behrouz Abedian

**Affiliations:** 1 Division of Pediatric Dentistry, Department of Preventive Dentistry, Faculty of Dentistry, Khon Kaen University, Khon Kaen, Thailand; 2 Department of Prosthodontics, Faculty of Dentistry, Khon Kaen University, Khon Kaen, Thailand; 3 Research Associate, Division of Biomaterials and Pediatric, Faculty of Dentistry, Khon Kaen University, and Dontan Hospital, Thailand; 4 Department of Restorative Dentistry, Harvard University School of Dental Medicine, Boston, MA, USA; 5 Department of Mechanical Engineering, Tufts University, Medford, MA, USA

## Abstract

**Background:**

Sealant application that yields superior marginal adaptation and deeper fissure penetration potentially improves success in preventive and restorative dentistry. This study evaluated the amount of in-vitro microleakage and penetration capabilities of different pit-fissure sealants as the effect of different application techniques.

**Material and Methods:**

160 freshly extracted human sound premolars, assigned as suitable for sealant application, selected and allocated randomly into 8 groups (n=20 teeth/group) and applied with different sealants including Embrace-Wetbond® (E), UltraSeal XT® (U), Clinpro™ (CL), Helioseal® (H), using either conventional (C) or induced application (I). The sealed teeth were thermocycling for 500 cycles between 5°C and 55°C with 30 seconds dwelling time. The tooth was coated with 2 layers of nail varnish, leaving 1 mm around the sealant margin, then immersed in 5% methylene blue solution for 24 hours. Subsequently, 2 pieces were segmented vertically in a buccolingual direction, yielding 4 surfaces/tooth for determination of microleakage and penetration proportion of sealant with polarized light microscopy (PLM) and image-J software. ANOVA and Bonferroni multiple comparisons were determined for significant differences (α=0.05). Sealant adaptability was detected using a scanning electron microscope (SEM).

**Results:**

The highest microleakage was observed for EC, followed by CLC, HC, UC, CLI, HI, EI, and UI. The highest penetration was seen in UI, followed by HI, CLI, CLC, UC, HC, EI, and EC. ANOVA indicated significant differences in microleakage and penetration on the type of sealant and application method (*p*<0.05). SEM revealed that the I-application method significantly promoted less microleakage and better penetration than the C-application (*p*<0.05).

**Conclusions:**

Microleakage and penetration capabilities of sealant are greatly affected by the types of sealant and the method by which the sealant is applied. U-sealant exhibited less microleakage and better penetration capability than others. I-application reduced microleakage, promoting enhanced penetration and adaptation, is the recommended sealant application.

** Key words:**Microleakage, penetration, dental sealant.

## Introduction

Dental caries is one of the most consequential infectious diseases known to humankind; however, dental caries in children and adolescents has declined in recent decades, because of improved preventive methods in developing countries. While smooth surfaces have benefited from caries-prevention protocols, the high prevalence of occlusal caries is still a problem (1). The main reason for this problem is the complex morphology of the pit and fissure on the occlusal surface of teeth, which are the most susceptible areas to developing caries. *Pi*ts and fissures are generally considered incomplete forms of enamel during the odontogenesis of a cusp. As a result, pits and fissures are narrow, deep, and irregular in morphology and they have been described as the single most important feature leading to the development of occlusal caries (2). The complex, irregular, and unpredicTable shape of these parts of the tooth favors the formation of caries and makes diagnosis difficult, complicated, and sometimes impossible through classical methods of assessment and diagnosis. Diets and dental plaque easily accumulate in these areas and cannot be removed effectively by the patient (3). Moreover, lack of saliva flow to the fissures and insufficient intake of remineralization agents do not compensate for a high incidence of occlusal caries (4). Modern dentistry has focused on preventive treatments such as systemic and topical fluoride administration. However, these methods preferentially protect smooth surfaces rather than occlusal surfaces (5). The structural defects of the occlusal faces are areas that favor plaque retention where fluoride is less effective. To prevent caries from developing in these zones, pit and fissure sealants have been developed successfully, and are used as an effective, minimally invasive preventive procedure nowadays (6). Fissure sealants isolate pits and fissures from the bacteria and their by-products, provide a mechanical barrier, and avoid an accumulation of dental plaque. The effectiveness of the sealant application is significant caries risk reduction compared to non-sealed controls and lower cost compared to restorative treatment (7).

Resin-based materials have been traditionally used as a pit- fissure sealants. Several types of resin, both filled and unfilled, with and without ﬂuoride release, have been employed. The main component of the resin-based sealant is bisphenol A-glycidyl methacrylate (Bis-GMA) resin. Resin-based sealants are generally used in combination with 37% phosphoric acid for etching, with appropriate moisture control can obtain good sealant efficacy (8). The resin-based material is lightly or not ﬁlled to keep the viscosity low, thus allowing for a deep penetration of the material into pits and ﬁssures, where a resin-impregnated layer of enamel is formed, producing effective sealing (9). The performance of pit and fissure sealant materials has been intensively investigated, yet no single product is reported as an ideal sealant. Glass-based sealants are principally recommended for pits and fissures sealing for two reasons. First, they are less susceptible to moisture which allows their use in non-cooperative children or in partially erupted teeth where isolation could be a problem (5). Secondly, due to their potential to act as a fluoride reservoir making enamel is more resistant to de-mineralization (10). In the case of difficult isolation, the use of glass ionomer cement is recommended (3). Some studies reported the use of bonding agents to enhance the bond strength of sealant to the tooth surface, especially in the case of saliva-contaminated surfaces, however, the technique is not widely used probably due to extended application time and increased cost (2,11,12). Recently, a hydrophilic resin-based pit and fissure sealant was introduced as a moisture-tolerant, self-adhesive sealant where the addition of adhesive bonding can be avoided. Several studies indicated that un-filled resin-based sealants exhibited better marginal sealing ability and bond strength to the enamel structure than filled resin sealants (13,14). Many studies demonstrated that resin-based sealants exhibited promising retention rates over glass-ionomer sealants because of their better stability under occlusal forces due to their main component, Bis-GMA (10). Conversely, some studies reported no significant difference in the sealing ability of filled-, unfilled resin, and fluoride-releasing sealants (15). Recently, a nano-filled resin-based dental fissure sealant has been introduced and claimed with ideal flowability with a concomitantly high filler content of up to 70 wt%. This sealant provides optimal wetting properties, high transverse strength, and excellent abrasion resistance. It was reported to have better microleakage characteristics compared to the filled sealant (16,17).

The efficacy of sealants strongly depends on their penetration capability into occlusal areas and maintaining an intimate adaptation of the sealant to the tooth surface (3,18). The marginal sealing ability of sealing material is extremely important for the success of sealants, which can be assessed by evaluating microleakage (19). Any breach in marginal integrity or weak sealing can lead to marginal leakage, resulting in a bacterial invasion, caries initiation, and progression underneath the restoration (7). Adaptation with adequate adhesion at the tooth-sealant interface is crucial for achieving clinical performance and durable restoration. Penetration of the sealant into the complete depths of pits and fissures and subsequent retain adaptation to the lateral wall of the fissure are also the key factors in the longevity of these restorations (4). An important step to increase sealing abilities is an acid etching of the enamel before resin-based fissure sealant application. Physicochemical interactions between sealants and acid-etched enamel are the principal forces providing sealant retention (20). Microleakage has been defined as the clinically undetectable passage of bacteria, fluids, molecules, or ions between the cavity wall and the applied restorative material (14). Microleakage studies are a standard method to access the sealant efficacy either in-vivo or in-vitro and can predict the marginal integrity of restoration and how successful they might last (6,16,17,21). Microleakage assessment may be qualitative or quantitative with different systems, including simple and computer-based methods. Dye penetration has been used in several studies to assess the presence of marginal leakage at the sealant-enamel interface (16). With the advantages of reliability, simplicity, and ease of application, the dye penetration test is a well-established and commonly used method for the determination of microleakage *in vitro*.

To increase sealant success, some attempts have focused on the procedure of modifying tooth surface before applying sealant, either invasive (enameloplasty) or non-invasive technique. The invasive technique either by enlarging a fissure with a drill or air abrasion was reported to enable better penetration and adaptation of the sealant compared to the conventional untreated fissures (18). The invasive technique also permits a better diagnosis of decalcification in the part nearest to the occlusal surface of the fissure, eliminates remaining debris, and increases the surface area for retaining the material. The preventive effect of pit and fissure sealing is mainly based on the ability of sealant materials to flow through pits and fissures and fill them without any gaps or air entrapments. As long as the sealant material remains bonded to enamel, effective protection will continue, providing longer-lasting and more successful applications (22). Accordingly, better marginal adaptation and deeper occlusal penetration are key in improving the rate of success of such treatment. In clinical practice, most sealants are often injected with a syringe onto the occlusal surfaces of teeth. For better adaptation, they are then shaped and spread around with a commercial applicator; however, upon withdrawing, the applicator leaves excess sealant which is light-cured. Evidence of placement difficulty with a commercial applicator for other dental sealants has been shown previously (23). This method of delivering sealant with an oscillating applicator will momentarily thin the sealant so that it will flow more readily without sticking to the applicator. It’s been shown that asymmetric rotating burs can provide superior marginal adaptation (24,25). However, the effect of a vibrating tool to control microleakage and sealant penetration using a micro-vibrating probe has been studied previously and no significant difference was detected (21,25). Other factors that influence microleakage between the enamel surface and the sealant include anatomical constrictions, the aprismatic structure of enamel, enamel surface preparation, moisture contamination, the application method of sealant, and the effect of temperature alteration. Thermocycling is the popular method to artificially induce aging of the specimens in the experimental study for evaluating the success of restoration upon temperature alteration. Sealant application technique is one of the main factors influencing the longevity of sealant. There still can be controversy in the effect of application technique on microleakage and penetration ability of different sealants. The purpose of this study is to compare the microleakage and penetration capabilities of various sealants with different application techniques. The null hypothesis was no significant difference in microleakage of various sealants upon application methods.

## Material and Methods

**Figure 1:**
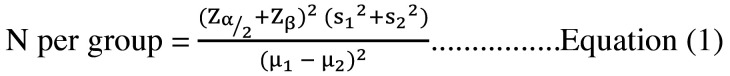
Formula.

An experimental study was conducted to investigate the effects of the application method on the microleakage and penetration ability of four pit-fissure sealants. The sample size for the *in vitro* study was estimated using the G*power 3.1 software (Heinrich-Heine-Universität, Düsseldorf, Germany) based on the statistical data from the Parco 2011 publication (26) using tests powers = 0.9, and α error = 0.05 as shown in Equation (1), (Fig. 1):

*Where: Zα = standard normal deviation = 1.96 (α error=0.05), Zβ = standard normal deviation = 1.28 (β error=0.1), µ1 - µ2 = mean difference between experimental group = 0.0121, and s = standard deviation (s1 =0.0049, s2=0.0144).*

**Figure 2:**
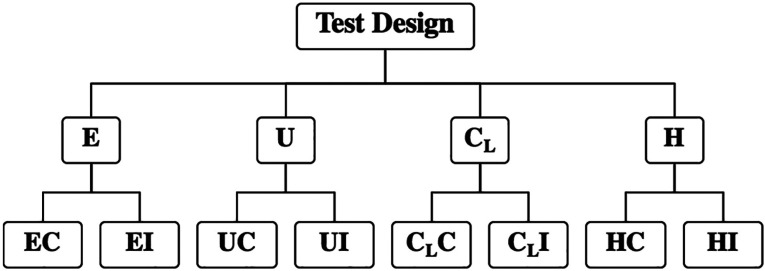
Consort flow diagram of the groups for the investigation of microleakage and penetration capability of four sealants [Embrace Wetbond (E), UltraSeal XT (U), Clinpro (CL), and Helioseal (H)] upon conventional (C) and induced (I) application.

The number of sample sizes based on this calculation was 20 samples per group used for this experiment. The investigation of microleakage and penetration capability of four sealants [Embrace Wetbond® (E)), UltraSeal XT® (U), Clinpro™ (CL), and Helioseal® (H)] upon conventional (C) and induced (I) application was designed as shown in the CONSORT flow diagram (Fig. 2).

-Sample preparation

**Figure 3:**
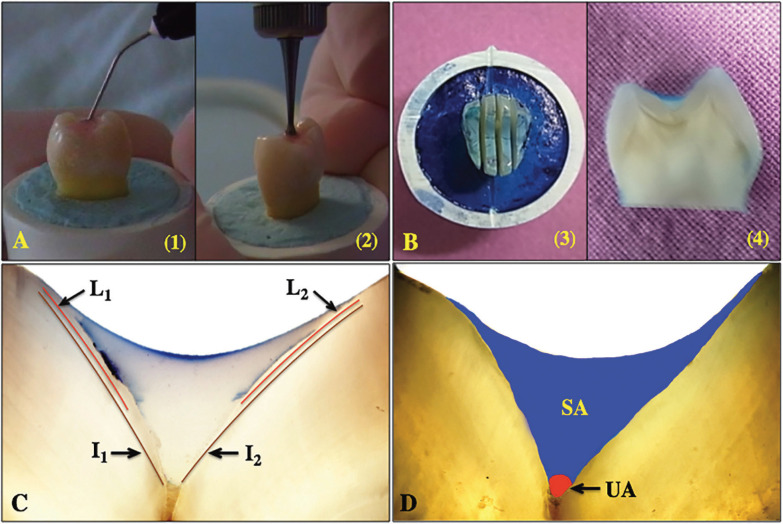
Placement of sealants (A) with conventional application technique and (1) induced application technique. Tooth-sealant was vertically sectioned (B) in vertical direction (3) to derive for disc sample (4). Microleakage (C) was determined from the length of leakage (L1+L2) per interfacial length of tooth-sealant (I1+I2). Penetration ability (D) of sealant was defined from the proportion of sealed area (SA) per unsealed area (UA).

**Figure 4:**
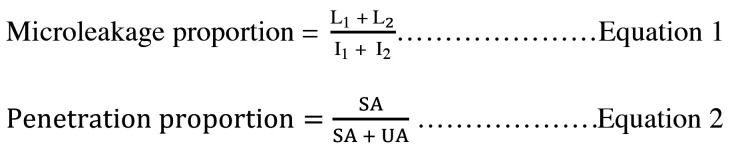
Formula.

A total of one hundred sixty freshly extracted human premolar teeth with no/or minimal occlusal caries for orthodontic reasons were stored in 0.1% Tymol (M-Dent, Bangkok, Thailand) in a dark container t room temperature and used for the experiment within  onths. Written informed consent was obtained from all the patients. All teeth were debrided at the surface with ultrasonic sealers and cleaned with non-fluoridated pumice using a rubber cup in a low-speed handpiece. Then, the root portion was embedded in the polyvinylchloride (PVC) resin ring and randomly divided into eight groups of twenty teeth each according to the type of sealants: Embrace Wetbond® (E, Pulpdent, Watertown, MA, USA ), UltraSeal XT® (U, Ultradent, South Jordan, Utah, USA), Clinpro™ (CL, 3M ESPE, Neuss, Germany), Helioseal® (H, Ivoclar Vivadent AG, Schaan, Liechtenstein), and techniques of sealant application: conventional (C) versus induced application (I). The occlusal surfaces of the samples were thoroughly cleaned for 20 seconds with a pumice slurry in a low-speed micromotor handpiece. The surfaces were then rinsed with air-water spray for 10 seconds and dried. The dried teeth were subsequently etched for 20 seconds with a 37% phosphoric etchant (Etch-RiteTM, Pulpdent), and rinsed for 10 seconds. The teeth to be sealed were air-dried but not desiccated. The sealant materials were applied according to the manufacturer’s direction using either conventional application method (C) using supplied cannula tip [Fig. 3A-(1)] or induced application method (I) using asymmetrical smooth round bur (Φ 0.8-1.0 mm) with slow speed handpiece (DIO SURGI CUBE, Busan, Korea) at a velocity of 1000 rounds per minute [Fig. 3A-(2)], and cured with visible light curing unit (Elipar 2500, 3M ESPE) for 20 seconds. After the application of the sealant, the samples were stored in distilled water at room temperature for 24 hours and subsequently aged in a thermocycling machine (CWB332R-MERL, KMIT’L, Bangkok, Thailand ). The thermocycle process was carried out for 500 cycles by soaking the samples in a water bath at 5°C for 30 seconds, resting at room temperature for 20 seconds, then soaking them in a water bath at 55°C for 30 seconds and resting at room temperature for another 20 seconds for each cycle (27). The surface of the tooth was covered with two layers of nail varnish leaving a 1-mm window around the margin of the sealant, and subsequently immersed in 5% methylene blue (Merck14279, Darmstadt, Germany) for 24 hours at 37°C, and then rinsed with distilled water. After rinsing, the specimens were fixed in a special cutting Table and then sectioned longitudinally in a buccolingual direction [(Fig. 3B-(3)] at low speed with a water-cooled diamond saw (Isomet 1000®, Buehler, Lake Bluff, IL, USA), thus providing two sections (1 mm thickness each) per tooth [Fig. 3B-(4)]. The surfaces of sectioned samples were polished with a silicon carbide abrasive up to # 4000 using a polishing machine (Ecomet®3 polisher, Beuhler).

-Determination of microleakage and penetration

After polishing, two sides of each sectioned sample were examined by a polarized light microscope (PLM, Nikon Eclipse LV100 POL, Shinakawaku Tokyo, Japan) at 40 X magnification, and photographed with a digital camera (Nikon D80; Nikon, Tokyo, Japan). The images were transferred to a porTable microcomputer to be analyzed for microleakage and penetration using image analysis software (Image-j, NIH, Bethesda, MD) after calibration to a millimeter ruler (9). One blinded examiner evaluated, analyzed, and recorded the depth of dye and sealant penetration in each section. After calibration to a millimeter ruler, the microleakage was evaluated quantitatively by measuring the length of dye penetration and the length of sealant penetration at the tooth-sealant interface. The microleakage was evaluated quantitatively in proportion by dividing the length of the dye penetration (L1, L2) by the length of the tooth–sealant interface (I1, I2) from two sides of each specimen disc, measured in mm (Fig. 3C), and calculated degree of microleakage using Equation 2. The sealant penetration capability was determined in the proportion of the area of sealant penetration per total area of the fissure. Likewise, the penetration ability was assessed as the proportion of the area of the fissure which is filled by the sealant relative to the whole area of fissure that the sealant should be filled (6) (Fig. 3D) as the Equation 3 (6,25), (Fig. 4).

*Where L 1 and L2 are the lengths of dye penetration, I 1 and I2 are the length of the sealant-tooth interface, SA is the sealed area of sealant, UA is the unsealed area of the sealant.*

-Microscopic examination

The surface of the samples was labeled and coated with gold-palladium at a current of 10 mA and a vacuum of 130 m-torr for 3 minutes using a sputter coater (K 500X, Emitech, Asford, British, England), then dried in a desiccator and finally evaluated the adaptation ability of sealant with the tooth surface along the tooth-sealant interface with a scanning electron microscope (SEM, Hitachi S-300N, Osaka, Japan).

-Statistical analysis

With an interval of a month, the observer randomly re-examined 10% of the samples. The intra-examiner reliability values of microleakage and unfilled area proportions using Spearman’s rank correlation coefficient were 0.95 and 0.98, respectively. The data of the microleakage and penetration capability were statistically analyzed for normality using the Shapiro-Wilk test. The mean and standard deviation (sd) of the microleakage proportion and the penetration capability proportion for each group of sealant materials, applied with different methods were calculated, compared, and then further analyzed using ANOVA in conjunction with post hoc Bonferroni multiple comparisons using statistical software (SPSS version 22, Chicago, IL, USA) to determine significant differences in the microleakage and penetration capability. The result was considered statistically significant at the 95% confidence interval (CI). The post hoc Bonferroni test was used to evaluate the differences between groups.

## Results

**Figure 5:**
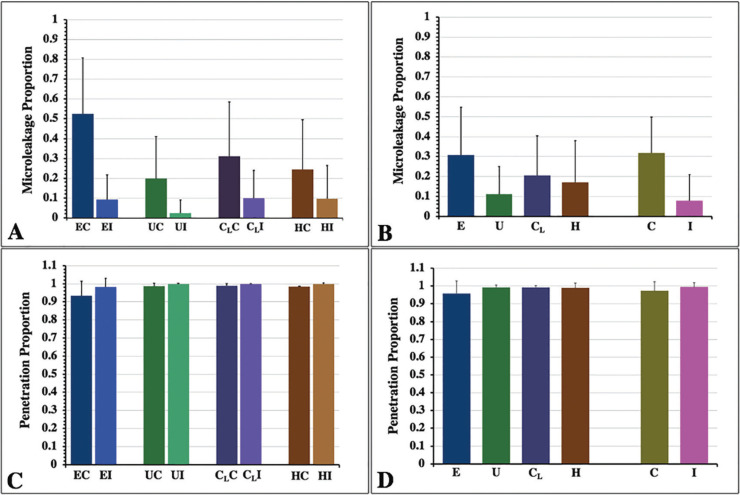
Microleakage proportion (A,B) and penetration proportion (C,D) of Embrace Wetbond (E), UltraSeal XT (U), Clinpro (CL), and Helioseal (H) sealants upon conventional application (C), and induced application (I) technique indicated the effect of sealants, application technique (B,D) and combination of both factors (A,C).

The mean, sd, 95% CI of the microleakage proportion, and the penetration proportion for each group are shown in Table 1. The mean±sd values of microleakage for each group were ranged from the highest to the lowest: EC (0.525±0.284), CLC (0.310±0.274), HC (0.244±0.254), UC (0.199±0.211), CLI (0.099±0.140), HI (0.097±0.167), EI (0.093±0.125), and UI (0.024±0.067), as presented in Figure 5A. The two-way ANOVA indicated a statistically significant difference in the microleakage of sealant with the application technique, the type of sealant, and the interaction effect between the types of sealant and the application technique (*p*<0.05), as shown in Table 2(A). Post-hoc Bonferroni multiple comparisons indicated that E sealant exhibited significantly higher microleakage than U, CL, and H sealant (*p*<0.05). Similarly, the CLsealant exhibited significantly higher microleakage than the U sealant (*p*<0.05). However, no significant differences in microleakage between H and U sealants (*p*>0.05) and between U and CLsealants (*p*>0.05), as shown in Figure 5B and Table 3 (A). The application method revealed a statistically significant effect on the microleakage of sealant (*p*<0.05), as shown in Figure 5B and Table 3(A). Post-hoc Tukey multiple comparisons indicated significant differences in microleakage between different sealants materials with the application method, except for the UI-EI, CLI-EI, CLI-UC, CLI-UI, HC-UC, HC-CLC, EI-HI, UC-HI, UI-HI, and CLI-HI groups, as shown in Figure 5A and Table 3(B).

**Table 1:**
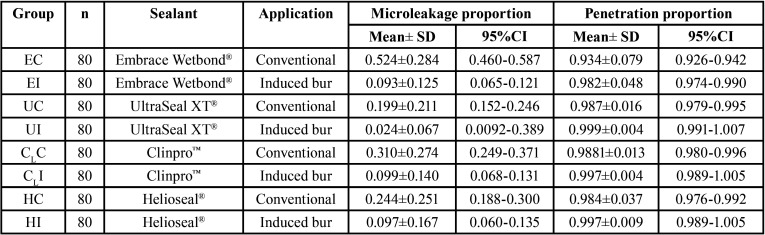
Means and SD of microleakage and penetration proportion of sealants sealed by different application techniques.

**Table 2:**
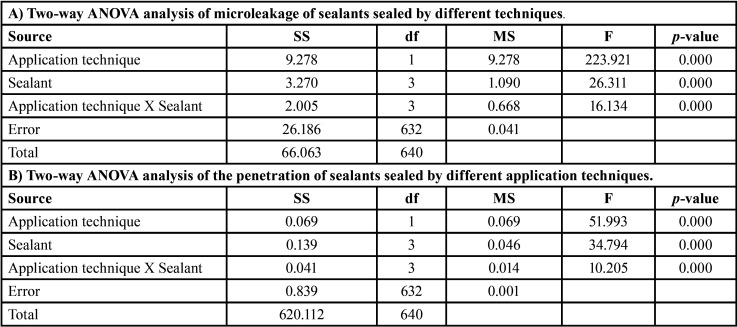
Two-way ANOVA analysis of microleakage and penetration of sealants sealed by different application techniques.

**Table 3:**
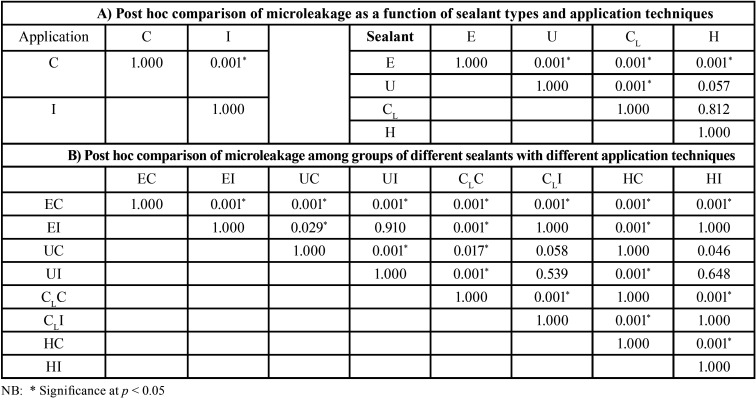
Independent T-test (A) and Bonferroni post hoc multiple comparisons (B) of microleakage of Embrace Wetbond (E), UltraSeal XT (U), Clinpro (CL), and Helioseal (H) upon conventional (C) and induced (I) application techniques.

The mean±sd values of penetration capacity for each group were ranged from the highest to the lowest: UI (0.999±0.0.004), HI (0.997±0.009), CLI (0.997±0.004), CLC (0.988±0.013), UC (0.987±0.016), HC (0.0984±0.037), EI (0.982±0.048), and EC (0.934±0.079), as presented in Figure 5C. The two-way ANOVA indicated a statistically significant difference in the penetration capability of sealant with the application technique, the type of sealant, and the interaction effect between the types of sealant and the application technique (*p*<0.05), as shown in Table 2(B). Post-hoc Bonferroni multiple comparisons indicated that U, CL, and H sealant exhibited significantly higher penetration capability than E sealant (*p*<0.05). However, no significant differences in penetration capability between U, CL, and H sealants (*p*>0.05) as shown in Figure 5D and Table 4 (A). The application method revealed a statistically significant effect on the penetration capability of sealant (*p*<0.05), as shown in Figure 5D and Table 3(A). Post-hoc Tukey multiple comparisons indicated no significant differences in microleakage among different sealants materials with the application method, except for between the groups of EC-EI, EC-UC, EC-UI, EC-CLC, EC- CLI, EC-HC, and EC-HI as shown in Figure 5C and Table 4(B).

**Table 4:**
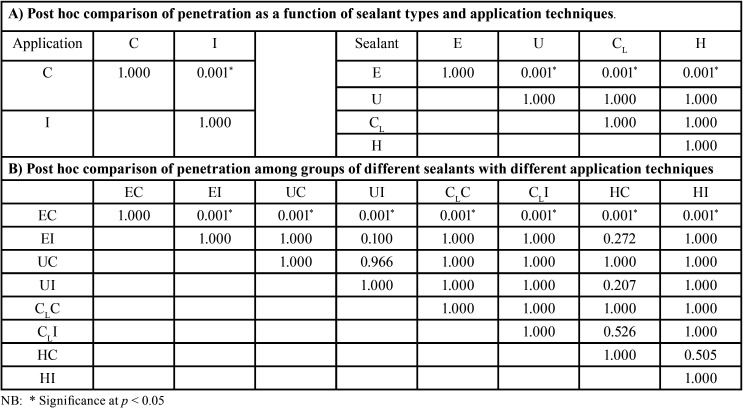
Independent T-test (A) and Bonferroni post hoc multiple comparisons (B) of penetration of Embrace Wetbond (E), UltraSeal XT (U), Clinpro (CL), and Helioseal (H) upon conventional (C) and induced (I) application techniques.

**Figure 6:**
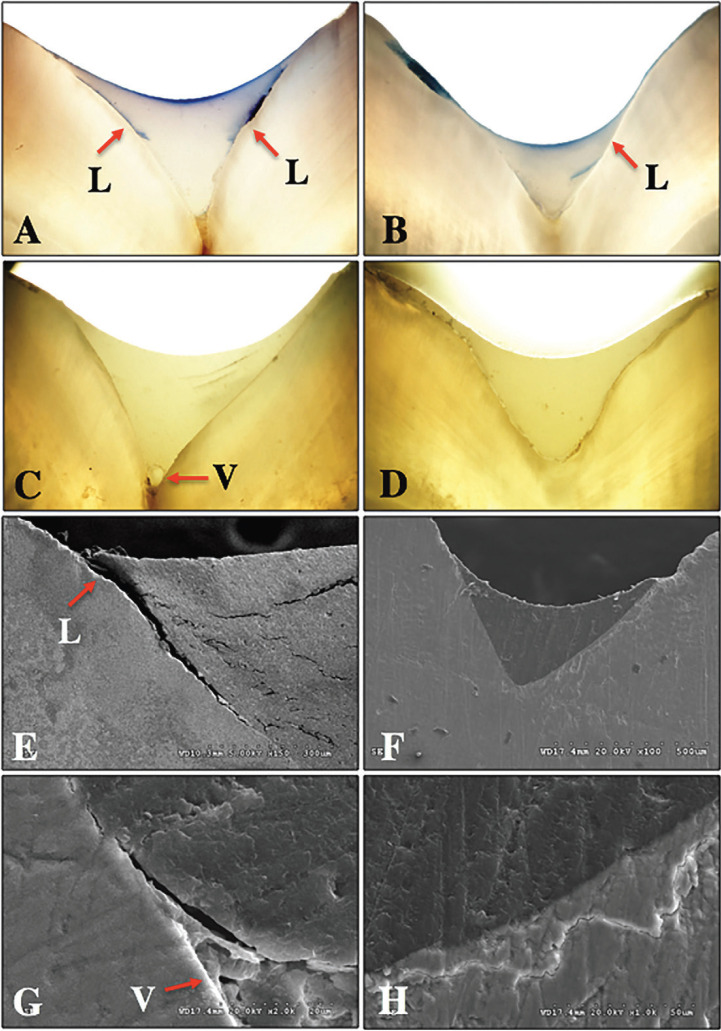
Polarized light microscopy (A-D) indicated microleakage (A,B) and penetration ability (C,D) of sealants upon conventional application (A,C) and induction application (B,D) technique. Different leakage (L) of sealant were indicated between application methods. Void (V) was presented at the apex of fissure for conventional application (C), whereas completely sealed was present for induction application (D). Scanning electron microscope photomicrographs (E-H) indicated larger microleakage at the margin (L) and the present of voids (V) at the apex of fissure of sealant upon conventional application (E,G) compared to induction application (F,H).

The polarized light microscopy (PLM) indicated that the sealant applied with the conventional application technique resulted in higher microleakage (Fig. 6A) than the induction application method (Fig. 6B). Moreover, the PLM indicated that the sealant applied with the conventional application technique resulted in less penetration capability (Fig. 6C) than induction application method (Fig. 6D). The conventional application method frequently presented with void (V) at the apex of the fissure (Fig. 6C), whereas completely sealed of the sealant into the fissure was presented for induction application, leaving no void at the apex of the fissure (Fig. 6D).

The scanning electron microscope (SEM) photomicrographs indicated that the sealant applied with the conventional application technique always presented a large gap at the border of the sealant (Fig. 6E), whereas no gap was presented at the marginal area of sealant applied with the induction application method (Fig. 6F). Furthermore, the SEM indicated that the sealant applied with the conventional application technique presented with less penetration capability (Fig. 6G) than induction application method (Fig. 6H). The conventional application method frequently presented with void (V) at the apex of the fissure (Fig. 6G), whereas completely adapted of the sealant into the fissure surface was presented for induction application, leaving no void at the apex of the fissure (Fig. 6H). The SEM photomicrographs indicated that the adaptation capability of sealant upon induction application method was better than the sealant applied with conventional application method (Fig. 6E-G).

## Discussion

The effectiveness of pit-fissure sealants depends on various conventional factors such as microleakage, penetration capacity, adaptation, bond strength, and viscosity of the sealant (28). The sealing ability of the restorative materials is the most important factor against microleakage (3). Sealing grooves, pits, and fissures is considered to be an important procedure among the strategies for preventing or reducing the risk of caries at the initial stages (18). Several studies reported that to reduce microleakage significantly, the sealing material must adapt perfectly (7,18,19). A better marginal adaptation and deeper penetration of sealant into the fissure is key in improving the rate of success of pit-fissure sealant, and this may be directly related to types of sealants and application techniques. This comparative study was carried out to determine the microleakage and penetration capabilities of various sealants applied with different application techniques. The results indicated that sealant application techniques significantly affected the microleakage and penetration abilities of different types of sealants. A significant difference in microleakage and penetration capability was indicated concerning the type of sealants and their interaction of material and application technique. Therefore, the null hypothesis was rejected for the application methods and types of sealants and their interactions. The microleakage of sealant was reduced as well as the penetration capability of sealant was increased upon the induced application method, compared to the conventional application method, which is in agreement with other studies (21,24,25).

The present study indicated that the induced application method by using asymmetrical round bur for applying a sealant at slow speed has much improved marginal adaptation with less unfilled fissure volume and much less microleakage compared with a conventional application method. The use of asymmetrical round bur for induced application in this study indicated a high percentage of reduction in microleakage for all tested sealants, ranging from the highest to the lowest: U (87.93%), E (82.25%), CL(68.06%), and H (60.24%). The induced application was capable of decreasing microleakage by 75.23% compared to the conventional application method. Likewise, the use of asymmetrical round bur for the induced application method in this study provided a significantly better penetration capability for all tested sealants, ranging from the highest to the lowest: U (92.36%), H (81.25%), CL(74.79%), and E (72.70%). The induced application was capable of increasing penetration capability by approximately 77.78% compared to the conventional application method. Thus, the authors are confident that in applying the sealant, a more successful sealant application with the induced application method. However, additional long-term studies are needed to demonstrate if such advantages may result in less failure when such induced applications are used.

The capability of induced application in reducing microleakage as well as increasing the penetration capability of sealants is probably related to several reasons. Firstly, the induced application rotating bur produced an oscillating effect on the sealant layer that resulted in thinning condition to sealant and better spreading of sealant into the pits and fissures easily as demonstrated by both PLM and SEM microscopy. The appropriate spinning speed of the bur led to the centrifugal force on the sealant that provide better adaptability of sealant to the tooth surface and decrease the sticking phenomenon of sealant with the bur as supported by other studies (24,25). The better adaptations of sealant to the tooth surface with the induced application are probably the ultimate reason for reducing the microleakage of sealant, ensuing in more successful sealant application. Secondly, the shape of the induced bur in an asymmetrical round shape of diameter 0.8-1.0 mm is capable of spreading, deforming, and manipulating the sealant much more effectively than a conventional application method and could be more effective in dental sealant placement as opposed to a conventional application (23,25). Thirdly, the induced application method provoked a vibrating effect on dental sealant, which are capable of enhancing the pseudo-plastic behavior of sealant, causing less viscosity and increasing the wetting ability to tooth surfaces with a better adaptation of sealant as supported in other studies (21,25). Conversely, if the conventional applicator was used to deform the sealant, the material often sticks to the plugger and any motion by the plugger was not capable of producing a better marginal adaptation. This study showed that better marginal adaptations with the induced application ultimately can reduce microleakage, resulting in more successful sealant application.

The favorable marginal adaptability of a sealant to enamel influences its efficacy and thus minimizes microleakage. Our study results showed that all the groups exhibited some degree of dye penetration. This finding is in accordance with other studies, that stated that microleakage can be expected in all restorative materials (8,29). This may be because the coefficient of thermal expansion of sealants (25-60 ppm/°C) is much greater than the coefficient of thermal expansion of the teeth, both enamel (11.4 ppm/°C) and dentin (8 ppm/°C). Therefore, to assess the *in vitro* performance of resin materials, thermocycling is one of the commonly used methods to simulate the long-term stresses to which the restorations are exposed. In this study, the temperature range was between 5°C and 55°C, which was claimed by various studies, to be the most clinically relevant (8,29).

Penetration depth is an important parameter that may increase the longevity of the sealant and affect the retention and adaptation of the sealant (1). The present study revealed a significantly increased penetration depth in the induced application groups than in the conventionally applied groups, so the null hypothesis was rejected. By the enlargement of the fissure entrance with enameloplasty, sealant material easily penetrates the fissures. The penetration-depth results of this study, in terms of application method, are consistent with previous studies (14,18,30). Material properties and the fissure morphology have a significant influence on the penetration ability of the sealants. Embrace WetbondTM is a silica-filled resin-based sealant comprised of di, tri, and multifunctional acrylate in an acid-integrating network, that is more viscous than conventional unfilled resin-based sealants, making the E-sealant penetration into the fissure depth more difficult (2). Insufficient penetration ability also makes them less retentive mechanically when compared with their opponents (4,18). Thus, the study results revealed that glass-ionomer-based sealants showed the least penetration depth with more microleakage, in harmony with previous studies (11). Theoretically, penetration is inversely proportional to viscosity. The higher viscosity may cause poorer adaptation of sealant to enamel and incomplete penetration to the bottoms of the pits and fissures, resulting in decreased retention. More fluid resins may penetrate fissures more deeply and spread more rapidly over the surface. Thus, an unfilled resin would be penetrated more deeply into the fissure system and, therefore, perhaps be better retained according to the results of previous studies (1,5). Regarding sealant penetration, UltraSeal XT® showed better penetration than others, which might be due to the combination of low viscosity of sealant that comprised of trimethylene glycol dimethacrylate (TEGDMA), di-urethane dimethacrylate (DUDMA), and methacrylic acid (MAA). These resin compositions made the sealant higher flowability, and less viscous (20). Thus, UltraSeal XT® exhibited less microleakage and better penetration capacity than others. In this study, Clinpro™ slightly better penetration than Helioseal® sealant, this is probably attributed to the lesser filler contained in Clinpro™ when compared to Helioseal®. The lesser filler loading contributes to low viscosity, the better penetration ability into pits and fissures as supported by another study (7). The resin matrix of Helioseal® consists of urethane dimethacrylate (UDMA), hydroxy ethyl methacrylate (HEMA) phosphate, and aromatic aliphatic urethane dimethacrylate for 70-80 wt%, combined with silicon dioxide, and fluorosilicate glass fillers for 15-25 wt%, which provide lower polymerization shrinkage due to long chain polymer, rigid monomer, ring aromatic bond not flexible. This result is consistent with the findings of previous studies (12,16).

The microleakage assessment in this study was done by storing specimens in 5% methylene blue dye for 24 hours (17). This method imparts perfect and easy visualization in the digital images for scoring with a clear reference point, thereby providing excellent contrast with the surrounding environment. The microleakage, penetration, and adaptation of sealant were evaluated with PLM which provides a means of direct visual observation of sealant materials to enamel walls due to its focusing in-depth and magnification. With the use of computer software for calculating the measurements, the results reflected results better than personal estimation using the scoring system.

## Conclusions

The efficacy of sealant is primarily related to the penetration capability of sealant to be applied with good adaptability to the tooth surface with persistent microleakage over time. Microleakage and penetration capability of sealant were affected by the types of sealant and application method. UltraSeal XT® provided the least microleakage and the best penetration capability, while Embrace WetbondTM produced the highest amount of microleakage and the lowest penetration capability among the tested sealant material. The method to apply sealant using the induced application technique is capable of decreasing microleakage and facilitating the penetration capability of sealant. To minimize the microleakage and enhance the adaptability of sealant to the tooth surface, the induced application technique is suggested for the sealant application process.

## Clinical implications

Pit-fissure sealants are used for the prevention of dental caries in vulnerable anatomic areas such as developmental grooves and deep occlusal fissures. The clinician better chooses a material bearing in mind the ideal properties that possess high penetration capability upon appropriate application method, provides excellent sealing ability, and good surface adaptation to resist microleakage for a period of service, which could translate into maximum clinical efficacy for the patients. Hence, this in-vitro study indicated that sealants possess different microleakage, penetration ability, and surface adaptability. The induced application technique is the simplest method that enables the reduction of microleakage and promotes penetration upon applying sealant and is suggested for day-to-day practice.
